# Understanding and learning from rural drug service adaptations to opioid substitution therapy during the COVID-19 pandemic: the What C-OST? study

**DOI:** 10.3389/fpubh.2023.1240402

**Published:** 2023-11-30

**Authors:** Jenny Scott, Hannah Family, Joanna May Kesten, Lindsey Hines, Josie Millar

**Affiliations:** ^1^Centre for Academic Primary Care, Population Health Sciences, Bristol Medical School, University of Bristol, Bristol, United Kingdom; ^2^NIHR Health Protection Research Unit in Behavioral Science and Evaluation, Bristol Medical School, University of Bristol, Bristol, United Kingdom; ^3^The National Institute for Health and Care Research Applied Research Collaboration West, University Hospitals Bristol and Weston NHS Foundation Trust, Bristol, United Kingdom; ^4^Population Health Sciences, Bristol Medical School, University of Bristol, Bristol, United Kingdom; ^5^Department of Psychology, University of Bath, Bath, United Kingdom

**Keywords:** rural, opioid substitution therapy, COVID-19, mental health, lockdown

## Abstract

**Introduction:**

In April 2020, in response to government COVID-19 advice, changes were made to the way English drug services operated. Methadone and buprenorphine were typically dispensed in 1- to 2-week supplies, and key working was conducted by phone/online. Previous studies have examined the impact of these changes on people from urban settings. This study adds the experiences and perspectives of people receiving care from drug services in rural areas and makes suggestions for future emergency planning.

**Methods:**

Telephone semi-structured interviews were conducted with 15 people receiving care in Somerset, Wiltshire, and Suffolk, rural counties in England. Reflexive thematic analysis was used.

**Results:**

Three overarching themes were found. “*Challenges of rural lockdown”* (theme 1) describes how rural community challenges, especially reduced or no rural public transport, were experienced. This hampered some OST collections, with consequential drug use. It also impeded connections to loved ones, worsening isolation. For participants who were struggling pre-pandemic, the intersection between this and their experience of revised drug service operations is embodied in “*Amplification of Social Disconnection: Cut off and unheard*” (theme 2). They felt a lack of support, particularly from remote provision key working. Participants who had supportive relationships and time in the pandemic occupied in ways they found meaningful, and others who struggled with anxiety or depression, found pandemic changes “*Fits better with my life”* (theme 3). They experienced more freedom for other things, gained support by other means, such as family, or felt more comfortable with remote engagement. A cross-cutting sub-theme “*Understandable Interruptions”* showed acceptance of pandemic disruptions.

**Conclusion:**

National guidance and organizational policy impacted participants in different ways. Those who had supportive relationships and occupied time were better able to make positive use of newfound freedoms and engage with community-level support. In contrast, those who had less stability, including mental health struggles and social isolation, felt cut off and unheard, particularly from key workers. Reduced rural transport was a significant community-level issue, which impeded OST collection and social support. We suggest emergency response plans be created for individuals taking account of their pre-existing personal situations.

## 1 Introduction

Increasing drug-related deaths in the United Kingdom (UK) are a public health crisis (1, 2). There is a network of drug treatment services across urban, rural, and small-town locations in the UK. Prescribing is undertaken by doctors, nurses, or pharmacists, with psychosocial support and case management provided by staff commonly referred to as “key workers” or “recovery workers”. Opioid substitution treatment (OST), such as methadone and buprenorphine, is prescribed to reduce opioid-related deaths and blood-borne viruses and support recovery from substance use (3). Recovery is defined as “*a process of change through which people improve their health and wellness, live self-directed lives, and strive to reach their full potential”* (3). Dispensing of OST is predominantly carried out by local community (retail) pharmacies. Compared to urban drug treatment services, rural drug treatment services are typically split over several locations across the area they serve, with fewer staff, who may have to travel between working locations, with the more widespread use of outreach. Rural pharmacies are typically smaller than urban ones, with less evening and weekend coverage. There are often challenges recruiting or retaining health and care staff in rural areas, which may have been impacted during the pandemic. Before the COVID-19 pandemic, national prescribing guidance meant many people received OST daily, sometimes consuming under the supervision of a community pharmacist, or in take-home installments of a maximum 1-week supply (4).

As a result of the public health measures introduced in the UK during the COVID-19 pandemic, there were rapid changes to drug treatment service operations. These were introduced because people who use opioid drugs were expected to be at increased risk of COVID-19 transmission and poorer health outcomes due to multi-morbidity (5, 6). Changes were designed to overcome potential barriers to treatment, reduce footfall in pharmacies and drug treatment services, and facilitate self-isolation. Changes included switching from face-to-face prescribing appointments and key working to telephone appointments, conducting of psychosocial groups online (e.g., via Zoom or MS Teams), and moving many people away from daily supervised OST consumption, instead providing take-home doses that covered longer time periods (7). Some services also switched people from methadone to buprenorphine as a risk reduction strategy and increased the speed with which patients were prescribed OST (8). Similar changes were implemented internationally (9).

UK studies conducted in urban areas (10, 11) or mixed urban and rural areas (personal communication) (8) during the pandemic suggest less frequent OST collection and relaxation of requirements for supervised consumption in pharmacies were generally viewed favorably by people in receipt of OST. Some experienced difficulties with managing large quantities of take-home OST or being pressured to sell or pass on their OST (8, 10). Remote prescribing and key working appointments were seen as convenient, but some found them less beneficial and impersonal, with a negative impact on well-being (8, 10, 11). The lack of in-person socializing brought about by online support groups is noted (10), while other studies (8, 11) report barriers to service access with subsequent loss of connectedness and isolation.

However, little is known about how people who use drug treatment services based in rural areas were affected by changes in pandemic service provision. Research is often focused on urban areas, even though 9.7 million people in England (17.1% population) live in rural areas. It is important to understand their experiences to inform future provision. In the US, the lack of mobile phone coverage in rural areas was a particular barrier to telemedicine (12). Further work from the US notes that rural areas can be subject to regional neglect and issues stemming from geographical isolation such as lower broadband coverage (13). Such regional neglect is also highlighted in England, in a 2022 Parliamentary Inquiry into rural health and care (14). It notes a lack of understanding of the health and wellbeing of people who live in rural England. It identifies healthcare inequities between rural and urban areas, describing poorer healthcare and worse health outcomes in rural areas, compounded by hidden deprivation and poor transport. Before the COVID-19 pandemic, rural transportation provision in England was already in decline (15). During the COVID-19 lockdowns, public transport was restricted further, and timings of services orientated around the working day or stopped altogether. The Parliamentary Inquiry (14) recommends delivering health services suited to the specific needs of rural communities. To do this, we need to first understand what those needs are. Kesten et al. (10) observed that public health measures implemented during the COVID-19 pandemic intersected with existing issues of poverty and isolation in the city of Bristol. We designed this sister study to Kesten's to explore whether people in rural areas had similar or different experiences to the urban studies already reported (8, 10, 11). Our study aimed to understand how changes made to the delivery of drug treatment services as a result of the pandemic response impacted on people who live in rural areas of England and to consider what we can learn for future pandemics or other emergency responses. This will allow approaches tailored to their needs to be developed.

## 2 Materials and methods

What C-OST? was a qualitative, cross-sectional, semi-structured telephone interview study carried out between October 2020 and April 2021.

### 2.1 Setting

Participants were recruited via seven English drug treatment services, in the counties of Wiltshire (South West), Somerset (South West), and Suffolk (South East). These counties cover large rural geographical areas. Recruitment was undertaken from services based in rural towns, as defined by the Office of National Statistics land use classifications. The services covered populations living in small towns, villages, and other rural areas. Services that served predominantly urban areas, as defined by the Office of National Statistics (Swindon in Wiltshire and Ipswich in Suffolk), were excluded. All services included in the study were part of one national third-sector organization that provides drug and alcohol services in these counties.

Data collection commenced in October 2020, shortly before a second national lockdown. The “*Everyone In”* scheme, launched in March 2020, gave funding to local authorities to provide rapid housing for homeless people in commercial hotels and hostels. This program became the Next Steps program in July 2020 with the addition of the Protect Program and Protect Plus Program for areas that needed additional support during lockdown restrictions in the winter. For the purpose of this study, we shall refer to accommodation provided to homeless participants as part of the COVID-19 response as “COVID-19 emergency accommodation”. Community pharmacies remained open during the pandemic, enabling people to continue to walk in without an appointment to seek advice, collect medication, or purchase items. They operated with staff and the public wearing masks, Perspex screens, and limits on the number of people entering the pharmacy at a given time. Some operated reduced opening hours, and they were permitted to close for staff breaks. Primary care (general practice) continued to operate, using a pre-booked telephone triage system. Drug treatment services included in this study had previously (pre-pandemic) operated with pre-booked face-to-face key worker and prescriber appointments, and in-person psychosocial support, which included group work. At the start of the pandemic, key worker appointments were switched to telephone, with group work initially paused and then introduced via online platforms. Prescribing reviews for people already on an OST prescription were switched to telephone. People prescribed OST for the first time, or the first time in that treatment episode, continued to be seen face to face with social distancing, masks, and other hygiene measures such as hand sanitizer. On the instruction of Public Health England (7) (now known as the UK Health Security Agency and Office for Health Improvement and Disparities), following risk assessment, most clients received 14-day supplies of OST from their pharmacy from April 2020. This was changed to 7-day supplies later in the pandemic. Pre-pandemic, a greater proportion of clients would have been on daily supervised consumption. No national data are published on supervised consumption levels, but a personal communication with one national provider advised that just over half of their OST clients were on supervised consumption pre-pandemic, and this figure is now around a quarter (2023), having been as low as 14% in the early pandemic phase.

### 2.2 Patient and public involvement

Four people with lived experience of drug use, drug treatment services, and OST gave feedback and comments on the study design and recruitment methods by telephone and online discussions with JS. In response to their comments, minor revisions to the topic guide were made to improve the clarity of the questions. They advised on an acceptable number of times to attempt to contact potential participants. Discussion with two drug treatment service managers confirmed that they considered the proposed methods (see 2.4) to be appropriate and realistic in the pandemic environment. Approval of the protocol was obtained from the management team at headquarters.

### 2.3 Ethics

Ethics approval for the study was obtained from the University of Bath School for Health research ethics committee (Reference: EP 19/20 061).

### 2.4 Recruitment and sampling

Drug service staff were gatekeepers to participants and supported recruitment. They were asked to identify opioid clients on their caseload who met the study inclusion criteria and give information about the study to them at their next telephone appointment. In the UK, the predominant illicit opioid used is heroin. Inclusion criteria were (i) pre-pandemic experience of drug service provision including OST from a rural service and (ii) 18 years or over. Ongoing feedback to gatekeepers attempted to encourage recruitment diversity. Those who expressed an interest in taking part and agreed to have their first name or nickname and telephone contact details shared with the researcher were then contacted by telephone by either HF or JS to arrange a convenient time. Those who agreed were sent a study information sheet (by post, or a link in a text or email). If the person was not contactable, HF/JS tried a maximum of four further times before their details were removed from the recruitment database. If the person declined to interview, their details were removed immediately.

### 2.5 Procedure

Interviews were conducted by JS and HF. As JS is a clinician at one of the study sites, HF engaged with and conducted all interviews with participants recruited from that service. Interviews were carried out by telephone because of the COVID-19 restrictions. When participants were called to be interviewed, the study was explained to them again, and the researcher made sure that they had seen the participant information sheet. Participants then verbally consented to participation and recording, which was done using a digital Dictaphone. The interviewer checked that the participant was somewhere where they were happy and safe to talk before commencing. At the end of the interview, the researcher debriefed the participants, and they were sent a £10 shopping voucher as an acknowledgment of their time given. Gift-giving audit processes did not permit the sending of cash without recording full names and contact details.

### 2.6 Interview topic guide

A semi-structured topic guide was developed to explore the impact of the pandemic on the individual and their drug use, on their experiences of drug treatment and service engagement, and take-home OST. It was based on the topic guide used for the LUCID-B study (10), which was developed by JK, LH, HF, JS, and other LUCID-B team members. At the start of the interview, participants were told “*We are interested to find out how the COVID-19 pandemic changes have affected people who use drug services who live in rural areas. This information will be used to help us understand how the pandemic has impacted on people like you and help us identify suggestions on ways services should be provided during and after the pandemic”* to preface the purpose of the study. They were also asked “*Would you describe your living location as countryside, village or town?”* to aid the use of appropriate prompts and support interpretation.

The topic guide and data interpretation were also informed by the socio-ecological model (16). This formed the theoretical framework for the study by using questions to explore the impact of government COVID-19 policy restrictions and Public Health England policy and resulting organizational service delivery changes on the individual and their interpersonal relationships/social support.

### 2.7 Data analysis and epistemology

Interview recordings were transcribed verbatim by professional transcribers and checked and anonymized by the interviewers. Reflexive thematic analysis (RTA) (17) was chosen because of its theoretical flexibility and was carried out by JS and JM who adopted a critical realism stance. There is one reality but that this is not independent of the researcher's perspective and their expertise and biases (18). As JS is a clinician and an academic whose research focuses on drug treatment and pharmacy services, she brought this knowledge and awareness to the research. She conceived the research idea with the knowledge that rural drug services and people who live in rural areas may have different experiences of drug services to those who live in inner city areas and that their experiences are often missing from the research literature. JS's practice experience of the service delivery model experienced by study participants informed analysis. JM is an academic and clinical psychologist whose research interests lie in the impact of loneliness and social isolation. JM also brought expertise in the method of RTA. Their joint expertise brought topic-specific and method-specific expertise to the coding and analysis. NVivo version 12 was used to manage data analysis. Data were coded inductively and framed around aspects of drug service provision, e.g., OST prescribing, dispensing, key working, and psychosocial support. JS and JM met frequently to discuss and refine codes, reflecting on the aim of the research, which led to some joint reorganization and renaming of codes. Once coding was complete, JM and JS met several times during the iterative process of code interpretation and theme building to reach a consensus. During theme building, we explored the impact of changes at different levels of the socio-ecological model on the individual and how, in turn, these changes altered their interpersonal and service provider interactions and relationships.

## 3 Results

### 3.1 Participants

Thirty-three people consented to contact, and 15 (eight women and seven men) completed an interview between October 2020 and April 2021. The remainder were mostly uncontactable after five attempts, or declined, for example, because they had changed their mind. The ages ranged from 31 to 56 years, with an average of 43 years. Table 1 summarizes demographic information about our study participants.

**Table 1:** Participant demographics.

**Participant code**	**Gender**	**Age**	**Self-defined rurality of place where currently living/sleeping (direct quote)**	**Accommodation during pandemic**	**OST medication**	**Pickup regimen at interview & (pre-pandemic)**	**Current OST prescribing (script) episode status**	**Experience of illicit opioids in pandemic?**
SR2	M	54	“a town”	Unstable/sofa surf, now in caravan.	Methadone	Weekly (daily)	On script before start of pandemic	Yes
W3	M	43	“Near town center”	Unstable/in car or tent, or with friends since lockdown restrictions eased	Methadone	Daily pickup, was on every 2 weeks (weekly)	On script before start of pandemic. Had restarted recently.	Yes
W7	M	54	“Small rural town”	Was homeless then in hostel now back with partner in house	Methadone	Weekly (daily)	On script pre pandemic. Started script again during pandemic.	Yes
W8	F	34	“Remote, 3 miles from town”	Living with family	Methadone	Every 2 weeks (daily)	On script before start of pandemic	Yes
W12	F	31	“Rural, outside town, no shops”	Housed (refuge)	Methadone	Every 2 weeks (weekly)	On script before start of pandemic	No
M15	M	38	“Very rural location”	Unstable/in car	Methadone	Every 2 weeks y (weekly)	On script before start of pandemic	Yes
M16	F	38	“Really rural, not much here”	Housed	Methadone	Was every 2 weeks and now weekly (weekly)	On script before start of pandemic	No
SF4	F	36	“a small town”	Housed	Buprenorphine	Three times weekly (daily) (declined weekly)	Started current script at start of pandemic	No
SF6	M	45	“Tiny little village”	Housed	First methadone/now Buprenorphine	Daily (weekly)	On methadone before start of pandemic. Was on reducing dose, detoxed in pandemic, relapsed, now on buprenorphine.	Yes
GB1	F	41	“Small rural town”	Housed	Methadone	Three times week (daily)	On script before start of pandemic	Yes
SF5	F	51	“a village”	Housed	Buprenorphine	Every 2 weeks and now weekly (weekly)	On script before start of pandemic	No
SF7	F	56	“Very remote…5 miles from nearest shop”	Housed	Methadone	Every 2 weeks (weekly)	On script before start of pandemic	Yes
STN2	M	47	“a town”	Was in a tent for 5 months, now housed.	Methadone	Every 2 weeks and now weekly (daily)	On script before start of pandemic	Yes
STN7	M	44	“Town center”	Housed	Methadone	Every 2 weeks and now weekly (weekly)	On script before start of pandemic	No
STN3	F	39	“Small town”	Housed	Methadone	Every 2 weeks (weekly)	On script before start of pandemic	No

### 3.2 Findings

**Figure 1:**
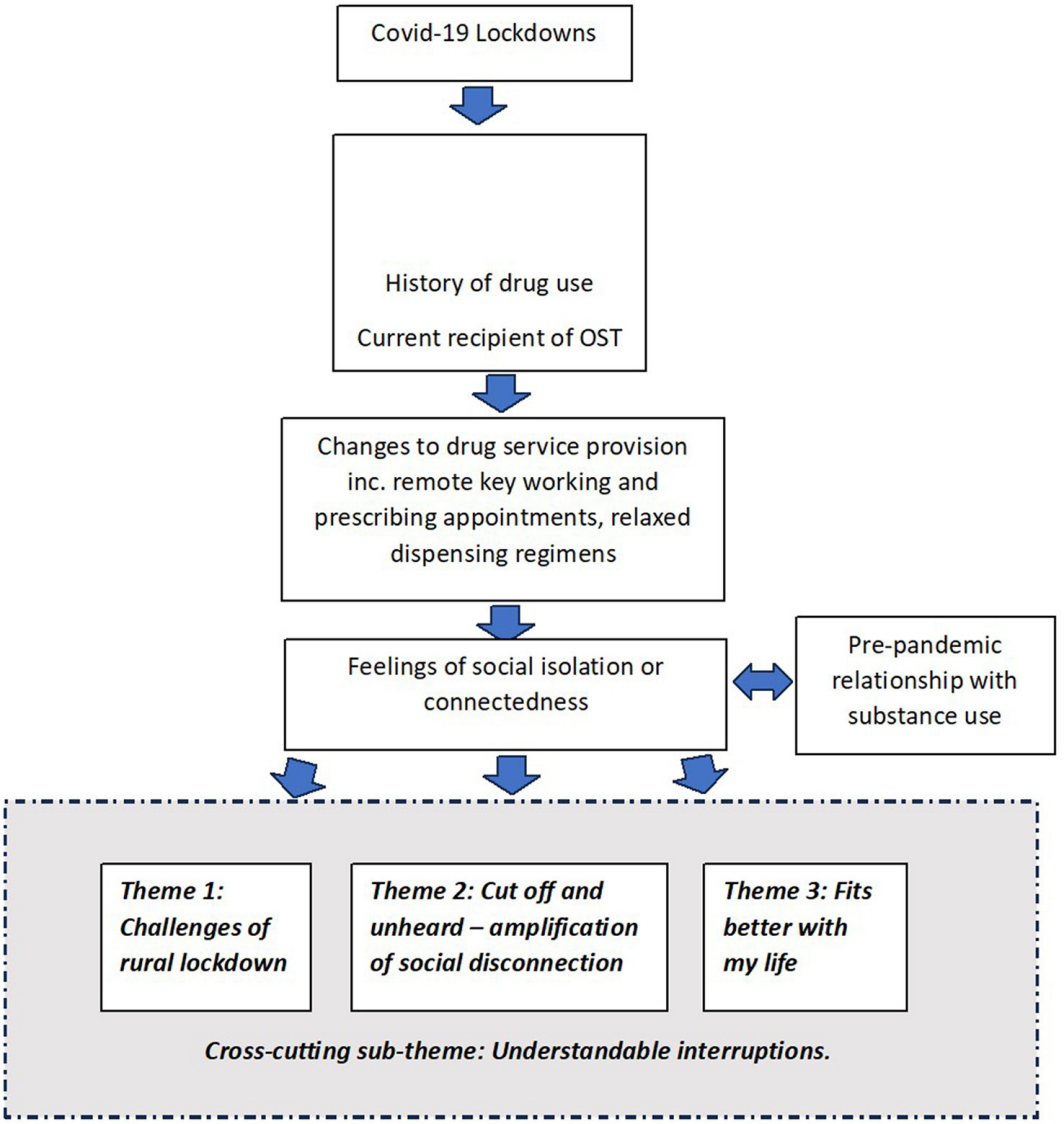
Conceptual model of the study findings.

Some participants had previously experienced a degree of social isolation before the pandemic due to the nature of their pre-existing difficulties with substance use. How participants experienced the changes in rural drug service provision was complicated by this pre-existing and/or current social isolation and influenced by their current relationship with substances. Three overarching themes were identified that depict experiences that were common across participants (as in theme 1) and varied among participants (as in themes 2 and 3). All participants had experienced being locked down in a rural area and pandemic drug services, including prescribing and dispensing of OST in rural areas. Theme 1 “*Challenges of rural lockdown”* encapsulates the difficulties experienced around this. For participants who were struggling with their recovery and treatment before the pandemic, the intersection between their experience of change in drug service operations and participants' pre-existing struggles resulted in an amplification of the latter, as described in Theme 2: “*Amplification of Social Disconnection: Cut off and unheard”*. For participants who had developed what might be described as more significant recovery capital [a term defined as “*the breadth and depth of internal and external resources that can be drawn upon to initiate and sustain recovery”* (19)] or who expressed less need for in-person support, the intersection was perceived to have a positive effect and is described in Theme 3: “*Fits better with my life”*. A cross-cutting sub-theme “*Understandable Interruptions”* ran through each of the overarching themes. Figure 1 conceptualizes the findings of this study.

#### 3.2.1 Theme 1: challenges of rural lockdown

Participants in this study had experienced significant transportation issues. They largely relied on buses for transportation. Some relied on public transport to facilitate OST collection. They described difficulties caused by infrequent services and how previous short journeys became multi-stage and lengthier or necessitated walking long ways. In some cases, this had contributed to missed collections as GB1 illustrates when describing the twice-a-day bus service from where he had been housed as a result of COVID-19 emergency accommodation to the pharmacy which he had started using previously when homeless:

“*I had to get the bus from where I was put* [housed] *and the buses only ran certain times of the day, so, and it was an hour and 15 minute journey on the bus to get there* [pharmacy] *and if I missed that bus, the last bus would be at 5:15pm and there wouldn't be a bus back, so I'd be stuck there…and I thought “no I'm not doing that”… I don't see why I should go to there and spend a night on the streets when I'm housed, that's not happening, so I have missed the odd day”*. (GB1)

One person who had no public transport available near their home had to rely on taxis. They reported being able to negotiate a metered fare rather than a fixed price due to regular use, although this remained a significant cost:

“*…it hasn't even got a shop here where I live, it's that small and I don't drive but it is just so lucky like there are taxi services….if the taxi services weren't on, I would have been “nah”* [to collecting OST in pandemic] *because I had to walk over a mile to get to my script…to walk it is like 40 minutes. But yeah, so I was just kind of lucky that some of the private taxi people were finding it hard in the pandemic, no one wanted to go to taxis, do you know what I mean? so I went out, I used them ….I got to know them and eventually they will just put it on the meter……the first time…they robbed me*, [like] *I was a tourist or something, I think it was twenty pound the first time and then it went down to twelve pounds”*. (W12)

Another participant explained how lack of public transport meant he could not travel for sterile injecting equipment which he did because he felt ashamed to ask for this at his OST pharmacy. Fear of using public transport in case of catching COVID-19 was mentioned by some. Some participants used pharmacies that had reduced their opening hours, which added to the logistical challenges of travel for OST collection. Others were able to walk to their pharmacy to collect OST so had not experienced public transport barriers in this regard.

Long queues at pharmacies, especially in the early weeks of the pandemic, had meant long waits for OST sometimes stretching to hours. Queues were often attributed to rural pharmacy staff being slow, or to restricted opening hours, rather than overt recognition of the large increase in demand for pharmacy services. Some reported being given priority within the queue because they were a regular client or because they had phoned ahead.

“*They are a bit slow, but I guess they are a small village bloody pharmacy and it is not quite fast pace there …. they are better now I phone them and say look I am coming in, they have it ready for me when I do that”*. (W12)

However, at times when the pharmacist had been absent (so no medication can be supplied), this had caused stress due to fear that they may not receive their OST.

“*Yeah there's been loads of queues at the pharmacy and stuff and sometimes there hasn't been a chemist there, so I'd have to go back and stuff like that, so yeah it's been quite difficult sometimes”*. (W7)

Participants largely spoke positively about less frequent pharmacy collections, reducing the stress around pandemic collection circumstances. As shown in Table 1, most participants were experiencing relaxed pickups once every 2 weeks or weekly. Unsupervised, less frequent pharmacy collection was seen as more “normal” (i.e., akin to how other non-OST medications are supplied) and less stigmatizing, giving the flexibility to take medication when preferred, such as at night time, or to split doses for comfort and reassurance. It brought reduced costs and less challenges from rural transport use. W12 illustrates the following:

“*I was able to pick my script up every two weeks, so it really…suited my needs, like, because, if I had to go every single day that would have…it was crazy, trying to get that* [pharmacy collection in early days of pandemic]*, but they understand that I am a mile away* [from pharmacy]”. (W12)

For some, waiting a long time in queues was made difficult by social anxiety, although less frequent pickups mitigated these challenges to some extent—it was bearable because it only had to be done once a week or every 2 weeks, as W7 shows:

“*Umm I suffer from anxiety and depression and I have bouts where I can't leave the house for months at a time so yeah the less I have to pick up the better really”*. (W7)

Others described putting up with the queues because of local convenience and the pharmacy staff treating them well. One person expressed concerns about being identified as a person in receipt of OST to others in his small town who knew him and who were also in the long queues. This centered around the use of indiscreet packaging:

“*All them people I live near* [in pharmacy queue]…*they* [pharmacist*] come out with your methadone in a clear bag, so all your neighbors can see it, instead of putting it in a brown bag and being discreet, yeah.”* (M15)

Appointments to start a prescribing episode or where there had been a break in treatment remained face to face, unchanged from pre-pandemic provision. Most participants had not experienced such an appointment during the pandemic. Telephone prescribing reviews, which were done quarterly, were generally well received and brought relief from the stress of navigating rural travel during the pandemic. The provision of other services remotely, such as key working and group work, was met with more mixed views and experiences, as embodied in themes two and three.

#### 3.2.2 Theme 2: “cut off and unheard”—the amplification of social disconnection

There was an intersection between social isolation, drug service operations switching to remote provision, and participants' pre-existing (pre-pandemic) mental health struggles, resulting in the amplification of the latter. Challenges with feeling unsupported, brought about by remote service provision included a sense of disconnection, particularly associated with telephone key working. Feelings of isolation were compounded by loss of social support, including from family and friends. Delays in being able to talk to keyworkers, for example, because of telephone answerphones, were also difficult in times of need. Participants felt unheard or as though they did not know what was happening, resulting in disempowerment. For some, this had led to or escalated substance use linked to worsening mental health. SF7 illustrates this:

“*I live on my own…the total lack of human contact really just being home alone 24/7, it's caused me mental health problems it really has…….Umm it hasn't helped me at all the lack of contact* [with drug services]*, and that, because when, as, my mental health deteriorated, the thought of using drugs was coming in, and when we were going through regular reviews and that, there's also a deterrent that we have, to give a test* [urine drug screen], *and so there was none of that. So I had nothing to stop me from using if I wanted to, and to be honest when, and I have used on and off through the periods of the lockdown….so it's been really unhelpful to me the lack of service* [drugs service contact] *and that. Really unhelpful….It was mainly the anxiety levels I just couldn't cope with them, being here on my own… feeling vulnerable and my anxiety just built and built and in the end I just felt I couldn't cope with life and obviously if you go and take some heroin, I know it doesn't make all your problems disappear, but it just puts you in a bubble sort of thing…. I've had depression on and off and for years but I've never had bad anxiety like that it, I just couldn't cope with it”*. (SF7)

Some participants expressed further frustrations regarding practical barriers to drug service access, such as not being able to walk into the premises when needed. Getting key worker support was difficult, with emotional consequences that impacted craving and thoughts about substance use compounded by staying home due to the pandemic. STN7 illustrates:

“*Yeah, because being, sitting there listening to music* [referring to being on-hold on the phone] *is like being in the dentist, it is not what you want happening if you are trying to give up drugs and alcohol, you want somebody to talk to, a face or a voice, just a bit of, you know… I can't think of the word but…just a bit of understanding rather than get wound up by going “these lot* [drug service] *don't care”. I have been waiting for hours* [for a call back] *and like I say, before you used to be able to walk straight in the office or phone them straight up and they would say you know, “What's up?” and you could get to talk to somebody…”*. (STN7)

Some talked of changes in their allocated key worker that had happened during COVID-19, resulting in not knowing who their key worker was, or not having met them, which amplified feelings of disconnect from the service.

“*Yeah I don't have a clue who my key worker is at the moment it's forever changing. You can't build up a relationship with someone unfortunately, cause yeah there's always different key workers…..that is a bit of a bummer …..I don't have that sort of knowledge and relationship with them like I've had previously* [pre-pandemic].” (STN3)

Some also talked of how it was easier to avoid engaging with services, as W12 illustrates:

“*Telephone- I am not going to lie, I have not been very good. Umm she* [key worker] *wants me to do like the group things* [online] *and that, and I haven't really said “no”. I said “oh I will try it” and then like I just haven't done it ….she hasn't bothered me again with it”*. (W12)

Disconnection was linked to digital poverty, e.g., lack of internet connection or IT skills. Unsurprisingly, given this study methodology, participants did not have difficulties accessing a telephone, but not all had mobile or smartphones. Some described others they knew with no phone access. Some found operating Zoom and Teams calls difficult, especially on phones. When discussing remote access to support, some talked of how impersonal telephone contact was and how much easier it was to open up and build rapport in a face-to-face key working session. GB1 and SF6 illustrate:

“*Umm I prefer face to face* [appointments with key worker]. *I mean, I've been into body language and psychology my whole life, so I like to be able to look people in the eye when I speak to them, “window to the soul”. You know what's going on when you look in the eyes. So when I speak to them* [drug service staff] *on the phone, I can just, all I do is listen to the tone of voice….But yeah I do prefer face to face”*. (GB1)

“*Just normal face to face human contact, conversation. I think when someone's doing their recovery, I think it's really important. I think they need that sometimes. I mean it's great ‘cause now we've got phones so they* [key workers] *Facetime and stuff, but it's not quite the same”*. (SF6)

#### 3.2.3 Theme 3: “fits better with my life”

Participants in this theme experienced helpful engagement with drug services and other sources of support during the pandemic. They found remote service provision suited their needs, gave more freedom, and described benefits to their mental health and well-being from this approach. They described ways to cope during the pandemic to mitigate, at least to some extent, their isolation, stress, and the challenges of rural lockdown described in theme 1. This included stability gained from employment or other daily tasks, examples given included gardening and looking after their house. They found that remote service provision aligned well with their needs and daily routine. For some, it was a preferred time-efficient alternative. People in this theme felt adequate support, some describing a parity between online and pre-pandemic face-to-face service provision. SF4 illustrates:

“*I think they've* [drug service] *been really good. I mean obviously we haven't been able to meet face to face. I've got a really good key worker, I haven't met him yet, but he's very good at keeping in touch and if I need him at all I can just text him and he'll ring me straight away, he's very good. We had a 40-minute appointment yesterday, so I think it's just been the same sort of thing as he would do it, but just over the phone”*. (SF4)

Some expressed a belief that not everyone needed the same level of support as their treatment journey progressed and that remote provision had allowed for this, compared to pre-pandemic service delivery where everyone was felt to be treated the same. STN3 illustrates:

“*Yeah it would be brilliant if they could* [keep remote service provision after the pandemic]. *Some people do need to go in, and at the start, when I first used the service, I did need that support, but as time goes on, I don't think it should be forced to have that full on support you need to start*, [you should] *be given a bit of space to get your life back”. (STN3)*

For others, they felt telephone appointments and online groups fitted better with their mental health, particularly anxiety, which for some had been worsened by pandemic circumstances. They described being able to participate and benefit from groups without leaving their home, something they had been unable to do or struggled to do pre-pandemic. W8 illustrates:

“*I had probably the most intensive contact with services than ever before, and actually I think it helped in a way, because had I been having to go to meet all these people face to face* [pre-pandemic groups] *and the way I was, with stress and anxiety and everything, I would have found just the thought of travelling and then speaking to somebody face to face, I think I would have found that really, really difficult. So the fact that I could just pick up my phone in my own environment meant that whole process* [was] *a lot easier”*. (W8)

The ability to be more anonymous and to have more control over their level of engagement in online groups was welcomed by some. STN7 illustrates:

“*Yeah, it's been really good yeah, we got sent out a workbook, it was an 8 week course with session 8 tomorrow, and you do a breathing exercise, umm, then introduce yourself to the group and talk about how your week has gone, and then you do, like, workbook based exercises and watch little online videos, have a little group chat and then umm, you basically don't have to be on there, you can keep your video off, so they can just hear your voice, so nobody is under pressure to even, you don't even have to say your name you can just put an exclamation mark next to your name you know? So, it has made people do it more, because of that, the fact that you don't have to tell people who you are”*. (STN7)

Some felt engagement with service providers by phone was enough for them. Engaging with prescribers was important and supportive, but key working and group work were not wanted. STN3 illustrates:

“*I did have the doctors, the prescribers, calling after the few months of them obviously getting a grip on what was going on, the doctors did start calling and they were fantastic. They were talking to us about mental health or talking to me about mental health, if you're having suicidal thoughts, all that sort of thing, but they were really, really supportive. So that was great because obviously it's quite good to talk to a prescriber, you can talk to a case worker but the prescriber is a doctor it just feels like they know a bit more, if you know what I mean”*.

She then goes on to expand on this point later in the interview:

“*I don't need that side of services much anymore* [group work], *I just need to make sure* [engagement with] *the prescriber if I need to talk to someone about my prescription. So I've done everything with the key workers, so there's not really much they could do with me cause I've done all the courses I've done. If I have a bad day I ring the Samaritans* [a national crisis helpline]”. (STN3)

Some talked of how the pandemic circumstance in general and the cessation of face-to-face attendance at drug services had helped them avoid coming into contact with others who use drugs, which was important to them to support their recovery. W12 explains:

“*They* [psychosocial groups] *helped me before, they did want me to get into it, I am not going to lie it is not for me……I find I don't want to hear about other people's horror drug stories and that, I have got enough of my own, do you know what I mean? Sometimes that can affect you, other people's stories, I don't know….like I think I just need to stay away from all of it, and since I have been staying away from all of it and not thinking of all of it, I feel better”*. (W12)

Some of these participants had also used drugs during the pandemic, but they tended to describe it as a “dabble” meaning it was a one-off or irregular for a short time, often triggered by boredom or fear that supplies would dry up. W8 explains:

“*At the beginning of lockdown I thought, I did have a few dabbles and I didn't need it, obviously, but it was kind of like, well what I'm not doing it nowadays, “‘oh my god what if there is suddenly a drought? I'd better do it now even though I'm not involved in that now” so it doesn't matter to me if there is a drought or not, but it was like the toilet roll and the shopping- pasta and rice and flour hysteria, it was like* [on] *mass, they're all doing it so I'd better do it”. (W8)*

These participants also experienced rural transport issues and physical isolation. However, they achieved connectedness by making adaptations to their lives, such as joining online activities or self-help groups, such as W8 and SF7 describe:

“*I was wanting to do like a little bit of exercise, for me, but when you're depressed you just don't want to move, and I went onto Reddit and I was talking to people in the depression forum and one of them mentioned that they played Pokémon Go. So you download it onto your phone and then the whole point of the game is that you explore your local community, so you visit cultural sites or historical sites, and they'd actually adapted the game during the pandemic so that you could play it socially distanced and build it into your one bit of exercise a day. And it was through that it got to the point where I was really looking forward to going for my walk, cause otherwise it would have been half an hour endlessly wandering thinking what's the point of this and probably over thinking everything and feeling worse”*. (W8)

“*Yeah I mean I do a lot of NA meetings on Zoom and stuff like that, I do one every day and have done for a few months, a Zoom NA meeting, to try and keep my head in the right place because that is the overall aim, to just reduce off the methadone and not be on anything”*. (SF7)

She later goes on to say,

“*Yeah I'm doing a basic computer course online, cause I got a laptop not long ago, and thank God I did, cause it's been worth its weight in gold. I mean, I could do the Zoom meetings on my phone but it's not the same sitting there holding your phone as having your laptop open in front of you. So yeah I'm trying to get computer literate and now sorted. I'm a bit more optimistic now coming out of this lockdown, so I've kind of started painting the house and stuff”*. (SF7)

Some altered living arrangements, or formed support bubbles, building on previously repaired or supportive relationships so tended to not feel so alone.

“*I see my two children* [in custody of her parents], [they] *haven't been at school through lockdown, so I've been round my mums everyday helping out there so that my mum and dad can still work”*. (SF4)

“*I see my step dad nearly every day and help him out in the garden, things to do now, his garden. Do a bit of work, he's quite active…Yeah and he can see his family”*. (SR2)

Keeping busy and maintaining normality through continued working were also coping strategies for some. Others such as GB1 kept busy and expressed a preference for being on their own:

“*Boredom very seldom touches me these days, I can read, I watch movies, I listen to music, I study, I write lyrics - I'm a musician as well, I've got plenty to do”*. (GB1)

In addition, a sub-theme of “Understandable Interruptions” ran across the themes. This demonstrates participant acceptance that disruption or interruption to “normal” service provision was inevitable, considering the size and scale of the pandemic. There was a recognition and tolerance of this. The extent to which it was felt services could modify their level of support, as opposed to the way they delivered support, was variable. Some felt that services had done all they could or done their best considering, and some felt that connections made by remote means could have been more frequent or done in a more supportive way. However, others had felt well supported, as described previously. SF7, who had previously completed the group work program face to face, felt she had been offered limited support in the early pandemic:

“*Umm only I know it was difficult for* [service name] *but I think they really need to find more ways of supporting people. If we get in this situation again, I don't think they can just kind of back off and leave us to do whatever, yeah, it's just not good enough. I know their hands are tied, they couldn't do face to face appointments and things, but I don't know why they couldn't have done some Zoom groups or something like that”*. (SF7)

Pharmacies were seen as doing their best considering the demands and restrictions. Two incidents of medication errors were described by separate people, due to wrongly measuring from multidose bottles. Participants largely described managing their medication and taking their doses with no untoward incident.

## 4 Discussion

This study has shown how people who receive care from rural drug services for opioid dependence experienced the support given to them during the COVID-19 pandemic, in response to Public Health England guidance (7), and how it impacted them in the wider context. We found that within their communities, participants experienced significant challenges around rural transport, as transport services were reduced or stopped, which made it more difficult to collect medication, and in one case, sterile injecting equipment. However, difficult journeys were mitigated to some extent by the need to only make them once every 1 or 2 weeks to collect OST. The adaptation of services to conduct prescribing review appointments and key working by phone also reduced the need for such journeys. Relaxation of service engagement requirements was experienced in one of two ways, which intersected with the extent to which the person had wider support or felt isolated. Those who described meaningful occupation of their time and connectedness, as described in theme 3, tended to be better able to make positive use of their newfound freedoms and also better able to engage with community support beyond the drug service, including online activities and family. We also found some who experienced anxiety were better able to engage with online support compared to in-person groups. In contrast, others felt cut off and unheard, particularly from key workers, as shown in theme 2. They tended to be those who described less stability in their lives at the onset of the pandemic, including mental health struggles and isolation from family. They struggled when trying to make contact with services or with knowing who to contact when they tried. This was contrasted with when it was easier to turn up at the premises and speak to someone face to face. The key worker–client relationship was seen as one of support and had an expectation of being able to discuss sensitive, personal issues, and of being responsive when needed. Some found this more difficult by phone. Infrequent contact from key workers had been experienced by some, across different services. This caused anger and frustration. Less frequent pharmacy OST pickups were welcomed, and pharmacies were mostly seen as having done their best considering the situation.

Many of our findings corroborate with other studies in the UK which have explored how people who use drugs have experienced the changes to drug service provision in urban settings. These studies also found a division in how participants experienced remotely delivered key working and group work (8, 10, 11). Those who had a positive experience acknowledged the benefits of less travel and greater flexibility. These authors also note some struggled to experience positive connections, reported greater isolation, and struggled with technology access. Kesten et al. (10) report how telephone prescribing appointments, less frequent pharmacy collections, and removal of supervised consumption were welcomed and seen as less stigmatizing, as reflected in our findings here. Similar to our study, they found those with poor mental health felt that isolation during lockdowns worsened their mental health. Schofield et al. (8) recruited participants from a mix of urban and rural settings in Scotland, another UK country, although no rurality distinction is made in their findings. We have shown similar experiences among participants who receive care from rural drug services to these previous studies (8, 10, 11) and suggest we found a greater emphasis among our participants on the challenges of rural transport, which as said was already in decline pre-pandemic (15).

Reflecting on international comparisons, Levander et al. (20) and Hoffman et al. (21) studied the impact of relaxed methadone take-home dosing in two rural opioid treatment programs in Oregon, USA. Both studies include findings that reflect those of our first and third themes. Both report greater take-home methadone dosing was welcomed because of the practical challenges and stress of rural travel in the pandemic and because of the greater freedoms that participants experienced allowing them to do other things, such as connect with family. Bolinski et al. (22) explored the experiences of people who use drugs in rural Illinois, USA. They found that some drug use escalated due to boredom and stress from the pandemic, which in turn amplified poor mental health, particularly depression. This reflects the experience we found in theme 2. Thakarar et al. (12) studied how the COVID-19 pandemic impacted on harm reduction services in Maine, USA (a rural state). They found benefits of remote (telehealth) prescribing appointments and outreach services during the pandemic and the provision of remote peer support groups overcame barriers of travel in rural areas. Their description of models of service delivery in Maine appears very different from those in England, so this may impede direct comparisons. For example, they report one-for-one needle exchange, which is not policy in England (23) and they report experiences of stigma from rural pharmacies, something not reflected in our findings.

Considering what we can learn from this study to inform future emergency response plans, we need to reflect on the approach that was taken in April 2020 across England in response to national advice (7). This advice and the consequent response of services are applied to both urban and rural settings. In future, rural issues could be considered at the national policy and guidance level and at the drug treatment service organization leadership level. The organization from which we recruited developed a risk assessment tool, which considered, among other things, the most recent mental health information, based on the person's last prescribing review or contact. This tool was used to assess risk for relaxed OST collection but not key working engagement. It was applied across the organization. Going forward, our findings suggest this tool could be reviewed, for example, to prioritize key working engagement for those with known current mental health struggles and/or social isolation as our findings showed some experiencing these issues wanted more support. We also suggest that an individualized approach is needed to decide the interventions offered going forward. Accounting for those who are more stable and want less contact and more freedom may be appropriate as our results in theme 3 suggest. Additionally, the tool should consider further local adaptations needed for people who use rural drug treatment services to take into account the difficulties amplified by the lack of transport reflected in Theme 1.

The dispensing of methadone into daily dose-measured bottles was advocated in national pandemic guidance (7) and requested on prescriptions from drug treatment services in our study. Some of our participants reported this had not been followed and had contributed to errors they made in dosing. This was also identified as a factor in a coroner's *Prevention of Future Deaths* report made during the pandemic in relation to a death in one of our study areas (24). We, therefore, want to emphasize to pharmacy staff, the importance of following such guidance and advising people on how to take and store their methadone safely. We recognize to do so when under the extreme work pressures of the early pandemic may have been difficult.

We suggest an online “engagement group” could be co-designed with people who use rural drug services, in readiness for future emergency needs. This engagement group could be specifically deployed to reduce isolation and maintain contact rather than deliver specific psychosocial interventions. Such a group could be a time and staff-efficient way to offer connection to those who would otherwise experience isolation. This could respond to the needs and suggestions identified in theme 2, where participants felt more contact was needed, although it may not overcome the sense of online contact being “not quite the same”. One of our participants suggested planning a buddy system bringing together lonely people which could be further explored.

In addition, depending on the nature of the emergency and government restrictions, it is suggested consideration is given to maintain outreach services in rural areas to connect with less stable clients and to ensure rural needle and syringe provision, e.g., by home delivery, as described by Kesten et al. (10) Bolinki et al. (22) found that such a model in rural Illinois, delivered with PPE and contactless, reached people during the pandemic who had not previously used the service and gave some much needed social support which they could not get elsewhere. Thakarar et al. (12) report collaborative working between organizations that support people who use drugs to facilitate rural harm reduction services. Such joint working during an emergency situation could be explored at the community level in rural areas in England, although the reliance of many on volunteers may be a barrier. We also suggest advanced thought is given to how to support people to access online offerings, both from the perspective of digital poverty and skills, and around their willingness and comfort to engage in this way. This is essential before any online engagement group, or buddy system, as suggested above, could be operationalized. Our findings also suggest more work is needed to understand, develop, and train staff on effective and acceptable remote key working delivery, for example, accounting for non-verbal communication cues in telephone appointments and trying to reduce the variability in remote key working experiences that this study found. We acknowledge we have not studied rural drug treatment service staff perceptions of remote key working or prescribing, which is a gap that needs to be part of future work.

Our findings suggest that although people experienced long pharmacy queues and some difficult journeys, these disruptions were accepted and mitigated by less frequent pickup. Participants in our study welcomed less frequent OST collection which felt more “normal” and less stigmatizing. When asked about consumption of medication at home, most had managed without incident and those reported can be mitigated as described. Consideration needs to be given to allowing greater flexibility in take-home dosing for people who are stable on OST going forward. Lister and Lister (25) note in rural parts of the USA, 28-day take-homes were given to patients deemed “stable” and 14-day take-homes for those deemed “less stable” but able to handle and store take-home doses safely. It is not clear how this was assessed. Those who have managed well on less frequent pickups should only be returned to more frequent dispensing if there is justification for the cost to the public purse and the inconvenience that this brings. When looking for such justification, we must consider the evidence to support the use of supervised consumption of OST, a practice which was started in Scotland in the mid-1990s when drug treatment services had long waiting lists, to reduce diversion. Ecological studies of supervised consumption have found an association with decreased methadone-related deaths (26), and other evidence indicates limited effects on retention, abstinence, mortality, or other adverse events (27–31). Furthermore, methadone diversion, which supervised consumption aims to prevent, may help people manage their drug use, prevent withdrawal, reduce hepatitis C risks, and develop social relationships (32). Analysis suggests that those prescribed methadone in England did not experience a greater number of methadone-related deaths during the first UK lockdown (March to June 2020), but conversely there was a large increase in methadone-related deaths among those not in receipt of prescribed methadone (i.e., taking non-prescribed methadone) (33). These authors found no significant increase for buprenorphine deaths in either group (prescribed vs. non-prescribed). Data for the subsequent months of the pandemic are yet to be published. Data make no distinction between rural and urban locations or supervised consumption status. A large ecological study is underway to understand mortality outcomes from the removal of supervised consumption during the pandemic (34) which is welcomed to inform future practice.

## 5 Strengths and limitations

This study shines a spotlight on the experiences of people in receipt of OST living in rural areas in the South of England, considering the intersection of individual- and community-level factors with national guidance and organizational policy. Previous study has focused on urban areas. This study echoes the findings of previous studies with added emphasis on the challenges people in rural areas faced, mostly around rural transport and the mitigation of these challenges, e.g., by less frequent OST collections and online groupwork. By showing these similarities, we bring the experience of people in rural areas into consideration of how people who use drugs and drug services experienced the COVID-19 pandemic changes to service provision. This study recruited people from a range of rural settings with over half the sample being women. By nature of the research we were not able to include people who did not have access to a phone, they would have been completely cut off from remote service provision. Their voices are not heard in this study which is a limitation. Considering previous UK studies, there are several similarities in findings, for example, mixed experiences of online service provision, a loss of connectedness with services felt by some and favorable views on lack of supervised dosing, and reduced OST collections (8, 10, 11). Challenges in undertaking research during a pandemic were faced. As experienced researchers in the field, we consider this recruitment to be more challenging than our previous in-person experiences. Online working caused difficulties in gaining visibility of the research team within services and to potential recruits. The pandemic circumstances meant services were under pressure and experiencing greater staff sickness, alongside the very rapid change in service delivery. Reliance on key workers as gatekeepers, most of whom were not known to us, impacted on our slow recruitment rates; it took 6 months to recruit and interview our participants. About half of the people who agreed to be contacted were recruited. Most who were not recruited were not contactable rather than they overtly declined. It was a difficult balance to try to deliver frequent gatekeeper reminders that did not feel like additional pressure, or to repeatedly try to contact potential participants without feeling we were harassing. Participants were recruited from one organization, so the way in which key working was delivered during the pandemic may have differed from that of other organizations although all services operated under national guidance (7). Finally, we must consider that our data were collected over a wider timeframe of the pandemic than the other studies that we draw upon (8, 10, 11) which may mean our participants had adapted to a greater extent to the “new normal” they were experiencing at the time.

## 6 Conclusion

The results from this study in rural settings reflected similar findings to previous studies in urban settings but with greater emphasis on the challenges from changes to public transport due to the pandemic. For people already struggling, the pandemic worsened their isolation and feelings of disconnection and impacted their ability to feel heard, particularly in their interactions with key workers. Those who felt more “on track” benefited from revised ways of engagement and less intrusion from the treatment system into their lives. Those who struggled with face-to-face engagement due to anxiety found it easier to engage with and benefited more from remote service provision. Pharmacy access was acceptable although difficult for some due to limited rural transport. This was mitigated by less frequent OST collections, which were welcomed. Considering future emergency situation response, we suggest involving clients in developing their own response plans and possibly online support models, within the permitted societal emergency response framework.

## Data availability statement

The raw data supporting the conclusions of this article will be made available by the authors, without undue reservation.

## Ethics statement

The studies involving humans were approved by University of Bath School for Health Research Ethics Committee (Reference: EP 19/20 061). The studies were conducted in accordance with the local legislation and institutional requirements. The ethics committee/institutional review board waived the requirement of written informed consent for participation from the participants or the participants' legal guardians/next of kin because telephone interviews conducted when COVID pandemic restrictions were in place.

## Author contributions

JS conceived the idea for this study, informed by her work as a pharmacist prescriber in a rural drug and alcohol service, and the Lucid-B study, led by LH, which JS worked on with JK and HF. LH, JK, and HF contributed their experience from LUCID-B. JS and HF undertook the What C-OST? study recruitment and interviews. JS and JM undertook the thematic analysis and led on data interpretation and conceptualization. JM developed the conceptual model of the study findings (Figure 1) and contributed her expertise from the mental health field. All authors contributed to regular study meetings, study design, interview schedule design, data interpretation, and writing of this manuscript equally.
